# Acute Isolated Thrombocytopenia Secondary to Meloxicam Use: A Case Report

**DOI:** 10.7759/cureus.21232

**Published:** 2022-01-14

**Authors:** Nardine Abdelsayed, Michael Frost, William R Betz

**Affiliations:** 1 Internal Medicine, Grand Strand Medical Center, Myrtle Beach, USA

**Keywords:** bleeding disorders, nsaids, isolated thrombocytopenia, drug-induced thrombocytopenia, meloxicam

## Abstract

Meloxicam is a widely used nonsteroidal anti-inflammatory drug (NSAID) that is used to treat pain and some inflammatory disorders. One rare side effect is drug-induced thrombocytopenia (DITP), which can result in hemorrhage and death if not treated urgently. This diagnosis can be missed if mistaken for other conditions such as ITP, which is problematic since the mainstay of treatment is medication cessation. We present a case of a 42-year-old Hispanic female who was recently started on meloxicam and presented with petechiae, ecchymosis, and a platelet count of 2 (normal 150-350 K/mm3).

## Introduction

Meloxicam is a commonly used NSAID that preferentially inhibits cyclo-oxygenase (COX) 2 as opposed to its counterparts which inhibit both COX 1 and COX 2. COX 1 plays an important role in gastrointestinal mucosal protection, as well as renal and platelet hemostasis. NSAIDs as a class are notorious for gastrointestinal (GI) [1], cardiovascular and renal side effects [2]. Due to its selective inhibition, this medication is associated with fewer GI side effects in comparison to its non-selective counterparts [3]. It has a high bioavailability at 89%, is processed by the liver and renally cleared with a half-life of 20-24 hours. A literature search with ‘meloxicam’ and ‘thrombocytopenia’ shows only two case reports associating meloxicam with DITP [4,5]. Thrombocytopenia in this setting may be severe and life-threatening. We report a case of meloxicam-induced thrombocytopenia presenting with severe petechiae, purpura, ecchymosis, and mucosal bleeding.

## Case presentation

A 42-year-old female presented to the emergency department due to widespread unprovoked bruising on her extremities and scattered petechiae that began the day prior. She initially noticed small petechiae which progressed to ecchymosis and eventually, mucosal bleeding. She had no prior medical history and took no medications besides meloxicam which she had started taking ten days prior for a ‘pinched nerve’. She endorsed no previous episodes of bruising or bleeding and reported no history of antiphospholipid syndrome (APS) or systemic lupus erythematosus (SLE). She had no known family history of any bleeding disorders. She denied melena, hematochezia, hematemesis, epistaxis, or other noticeable signs of bleeding. In the emergency department, she was found to have severe thrombocytopenia. 

**Figure 1:**
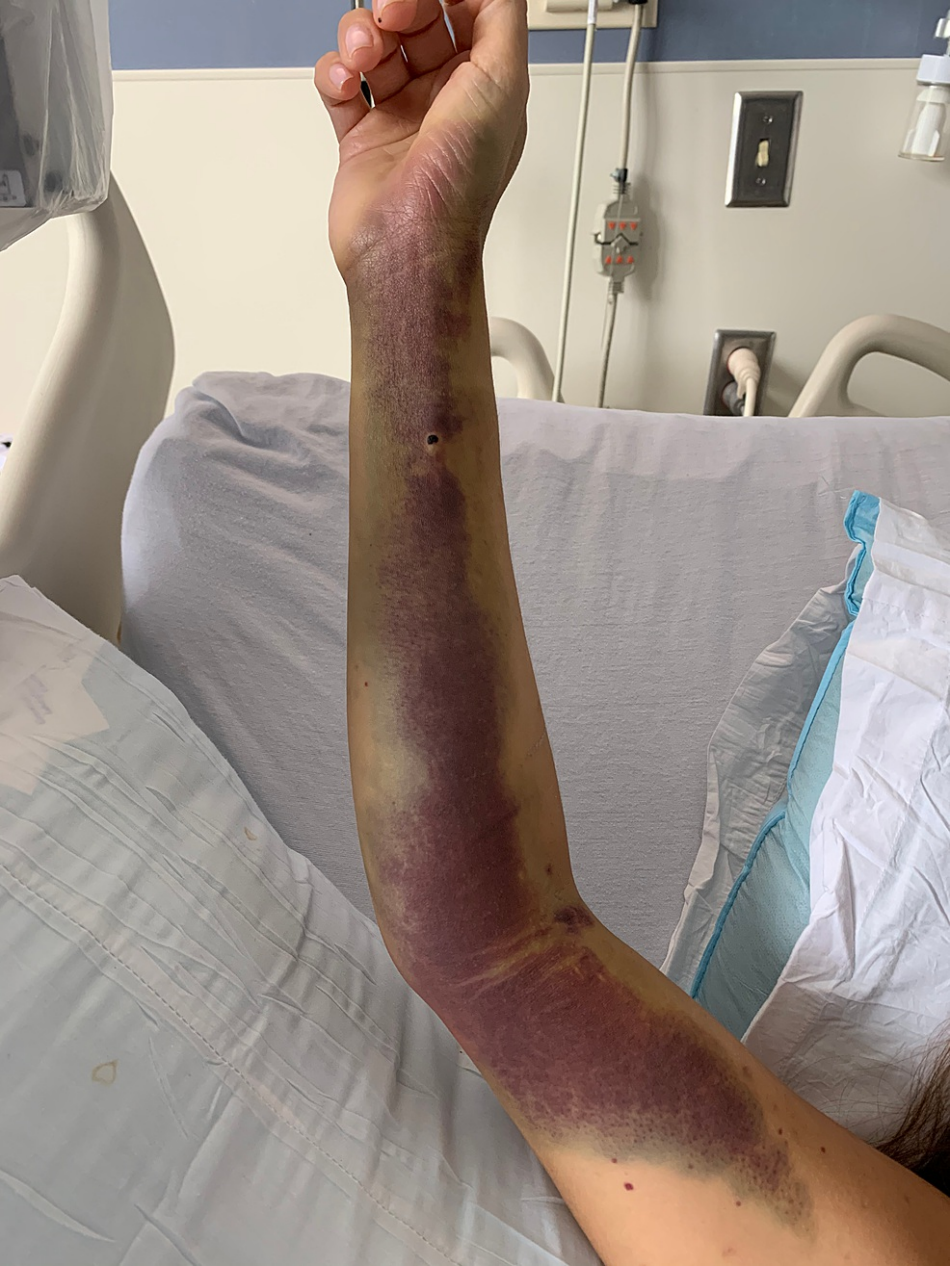
Extensive ecchymosis on the right upper extremity

**Figure 2:**
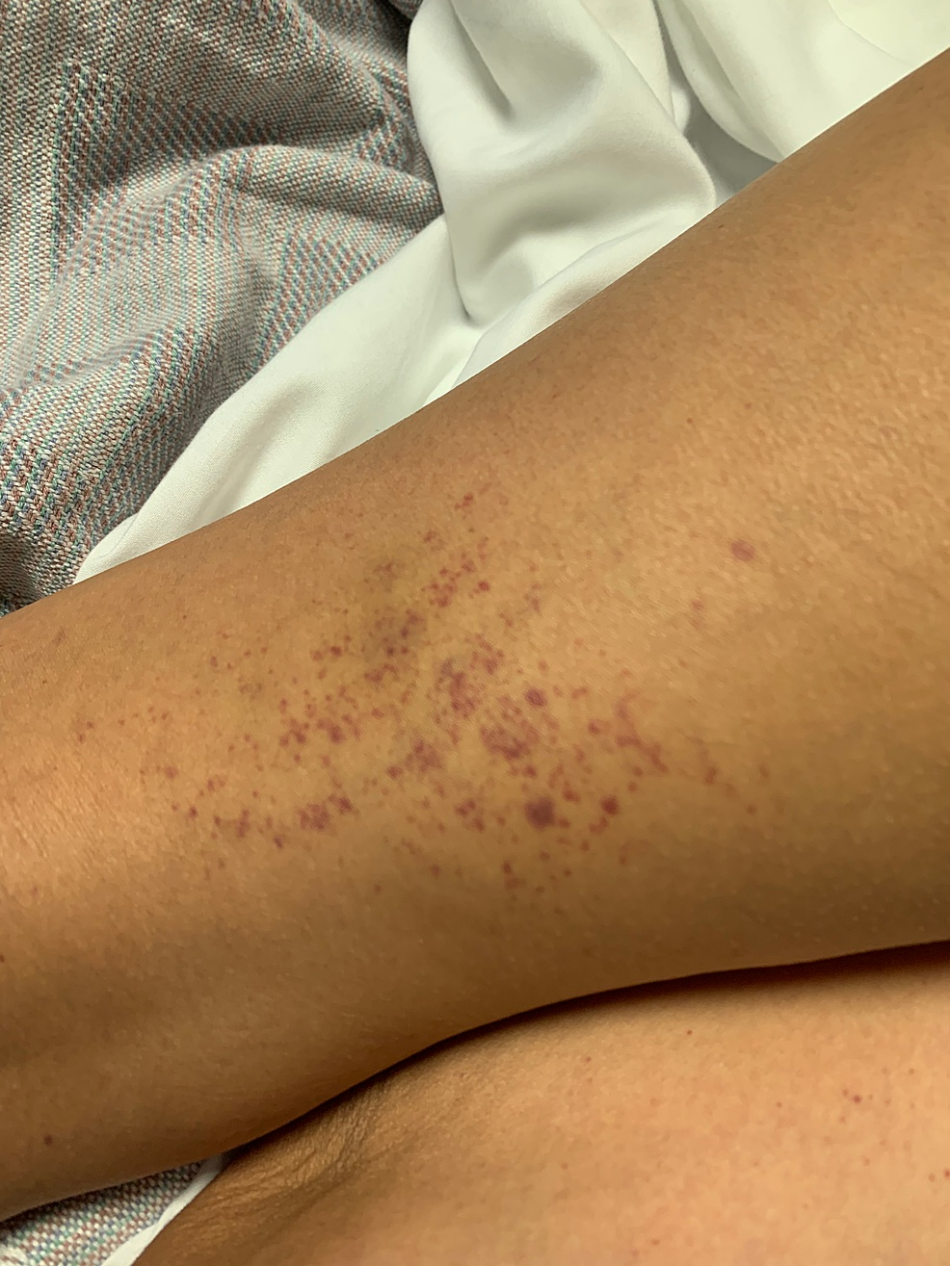
Lower extremities showing petechiae and purpura

Vital signs on admission were within normal limits with physical exam only notable for ecchymosis on her extremities (Figure 1), as well as petechiae on both legs (Figure 2) and oral mucosa. She had no palpable splenomegaly or lymphadenopathy.

Complete blood count (CBC) was notable for a platelet level of 2 K/mm3 (normal 150-350) on admission and other cell lines were within normal limits. Comprehensive metabolic panel (CMP), prothrombin time (PT), and international normalized ratio (INR) were all within normal limits. Her peripheral blood smear showed thrombocytopenia and was negative for schistocytes. Coronavirus, human immunodeficiency virus (HIV), and hepatitis C were all negative.

Meloxicam was discontinued. Due to the differential of idiopathic thrombocytopenic purpura (ITP) and severe thrombocytopenia, the patient was started on high-dose steroids for three days without any improvement in her platelet levels. Hematology was consulted and the decision was made to start intravenous immunoglobulin (IVIG) for a total of three days. Finally, on the third day of receiving IVIG, the platelet count rose to 34 K/mm3 with substantial improvement in her mucosal bleeding and petechiae.

At that time the decision was made to safely discharge the patient to return home with strict instructions to follow up with her primary care provider for further care. She avoided NSAIDs and aspirin going forward. Her platelet count eventually rose to a normal value and have remained normal at one-year follow-up.

## Discussion

Drug-induced thrombocytopenia (DITP) may occur by either immune-mediated platelet destruction, or by a nonimmune dose-dependent bone marrow suppression and subsequent decreased platelet production [6]. Immune-mediated DITP is commonly caused by drug-specific antibodies that bind to platelets, usually resulting in severe thrombocytopenia about one to two weeks after initiation of this new medication. On some occasions, thrombocytopenia may occur immediately after a single dose administration of the drug. Notably, the thrombocytopenia is only sustained by the continuation of the drug, and discontinuation of the medication should allow for the return of normal platelet counts [7]. DITP is commonly misdiagnosed as idiopathic thrombocytopenic purpura (ITP) and treated as such, sometimes to the extent of splenectomy [8]. In some cases, patients have thrombocytopenia that recurs whenever patients re-initiate the inciting drugs. Return of normal platelet count on drug cessation, and recurrence of thrombocytopenia in the presence of the drug are hallmarks of DITP when other etiologies of the thrombocytopenia are excluded [7]. 

Although the treatment for DITP is simply the cessation of the inciting medication, in many cases this disease cannot be easily differentiated from ITP and a course of steroids is initiated. If the cessation of the medication causes a return of platelet counts, it is appropriate to then stop steroids. In the case that platelets begin to decline once more, ITP may be more likely and steroids should be re-initiated. Since antibodies to the drugs in question remain for years, patients should be counseled to avoid this class of medication indefinitely, including aspirin in this case due to structural similarity [4].

In our case, discontinuation of meloxicam was an important step in management. Generally, platelet count begins to rise at about 4-5 half-life of the association drug [9]. Meloxicam has a half-life of about 20 hours [7] and thus, platelets would be expected to rise around 3-4 days after cessation, although rarely platelets may take weeks to recover [10]. Our patient's platelets began to trend up on day seven (Table 1), which is longer than usual but has also been seen in some cases of DITP.

**Table 1:** Platelet count during hospitalization

Hospital Day (Days)	Platelet Count (K/mm3)
1	2
2	2
3	1
4	1
5	1
6	2
7	2
8	8
9	32

Our case highlights a side effect of the commonly used drug meloxicam. In the case where severe thrombocytopenia is seen two to three weeks following initiation with no other probable cause, meloxicam should be urgently discontinued and the patient should be counseled to avoid this medication in the future. 

## Conclusions

Overall, we conclude that although rare, meloxicam may be associated with severe, potentially life-threatening thrombocytopenia and is an important component of the comprehensive history for acute isolated thrombocytopenia. Knowing this side effect of meloxicam leads us to the most important step in treatment, which is medication cessation. Otherwise, the thrombocytopenia will persist leading to grave morbidity and mortality. 
